# Geographic Variations in Intertrochanteric Femoral Fractures in China

**DOI:** 10.1155/2019/8396723

**Published:** 2019-10-17

**Authors:** Qian-Hao Yang, Yi-Xuan Chen, Dao-Yu Zhu, Zi-Sheng Ai, You-Shui Gao

**Affiliations:** ^1^Department of Orthopedic Surgery, Shanghai Jiao Tong University Affiliated Sixth People's Hospital, Shanghai 200233, China; ^2^Department of Medical Statistics, Medical College, Tongji University, Shanghai 200092, China; ^3^Centre for Orthopaedic Research, Faculty of Health and Medical Sciences, The University of Western Australia, Nedlands, Perth 6009, Australia

## Abstract

**Background:**

Hip fracture is one of the major risk factors of global mortality and disability. The aim of this study was to map the pattern of intertrochanteric femoral fractures in China, providing a pilot national dataset and basis for medical policy proposals.

**Methods:**

A multistage probability sampling strategy was applied in the national baseline survey. Thirty provinces in mainland China were included in this survey. A standardized questionnaire survey was conducted to collect information about basic characteristics such as age, working seniority, hospital level, and residence, with two other parts including perioperative and postoperative treatment parameters. Odds ratios and 95% confidence interval were used to determine essential statistical differences. The proportion of the options in each region was compared using the chi-square (*χ*^2^) test. The histogram and choropleth map of the monthly number of admissions were created using Excel 2016 to show the distribution characteristics.

**Results:**

In total, 1065 valid responses were included, representing a 96.7% survey capture rate. Perioperative treatment and postoperative care distinctly varied across regions and hospital levels. The monthly number of admissions was relatively lower in the Northern region, with higher proportion of hospitalizations to secondary hospitals compared with the Eastern region. The patients in the Eastern region or tertiary hospitals had shorter preoperative waiting time and hospitalization period.

**Conclusions:**

We found apparent geographic variations in intertrochanteric femoral fractures in this study, and the data can be used for drafting national healthcare plans and medical policies.

## 1. Introduction

Hip fracture is one of the major risk factors contributing to global mortality and disability and results in marked health and medical insurance burdens [1, 2]. The worldwide incidence of hip fractures is projected to increase to 2.6 million by 2025 and reach approximately 4.5 million by 2050 [3]. In China, the increasing aging population and changes in lifestyle related to urbanization have led to a significant increase in the incidence of hip fracture [4–6], while it gradually decreased since 2002 in the United States [7]. Hip fractures have poor prognosis, with a 1-year mortality of approximately 20%–30% [8–10]. Patients may not be satisfied with the level of function despite survival [11, 12]. The absence of intermediate care and prophylactic treatment highlights the undertreatment of this population.

The total population of China is projected to decrease until 2050, but a sharp increase in the proportion of the population aged over 65 years is expected. In general, 90% of hip fractures occur in patients aged 65 years and older [13]. The types of hip fractures have also changed over the past decade. The number of unstable extracapsular fractures, such as intertrochanteric and subtrochanteric hip fractures, increased rapidly while the number of intracapsular hip fractures remained unchanged [14]. Intertrochanteric and intracapsular femoral neck fractures are the main types of hip fractures in the elderly. Although both fractures share considerable similarities, they have different susceptible populations and potential risk factors [15]. Patients with intertrochanteric fractures are older and therefore have more comorbidities and higher risk of postoperative mortality [16, 17]. Moreover, the disease burden of intertrochanteric fractures is heavier in China [1, 18], and thus, medical policy with the consideration of geographic variations is urgently needed.

However, national epidemiological data for intertrochanteric fractures are limited to date. By contrast, there are several population-based observational studies on hip fractures in developed countries [5, 16, 19]. Previous studies have identified geographic variations in hip fractures, with accessibility/remoteness, socioeconomic status (SES), genetic background, racial difference, and climatic circumstance being major influencing factors [7, 20–22]. A recent epidemiological study in Shanghai reported an average age of hip fracture patients of 82.20 years, lower male-to-female ratio, multiple comorbidities, and severe complications [17]. However, geographic characteristics of intertrochanteric fractures across the Chinese population have not been elucidated. With the 1.38 billion population and the significant diversity in socioeconomics, lifestyle, hospital facilities, and healthcare policy, China is a vast country by which research from other countries can hardly be applied [23]. The aim of this study was to map the pattern of intertrochanteric femoral fractures in China, providing a pilot national dataset and basis for medical policy proposal. Towards this goal, we designed the national investigation of hip-fracture care (NIH-FC) questionnaire survey.

## 2. Materials and Methods

### 2.1. Participants and Protocols

The NIH-FC is a national orthopedic surgeon-oriented survey conducted by our research team. Because there is an immature disease registry system in China, it is impossible to employ a database methodology to reflect the comprehensive temporospatial characteristics of hip fracture care in China. As such, the authors endeavored to employ a survey-based approach. To determine the geographic features of hip fracture care, we designed a questionnaire to investigate the baseline characteristics of hip fractures. The subjects enrolled in this survey were registered orthopedic surgeons to minimize heterogenicity and improve efficiency [24].

In brief, a multistage probability sampling strategy was applied in the national baseline survey. Thirty provinces, including municipalities and autonomous regions in China, were included in this survey. Considering the difference in SES and climate, the provincial capital city was selected in each province. For the hospital-level sampling, 3 secondary hospitals and 3 tertiary hospitals were selected in each city using the probability proportional to the size method [25]. In China, secondary and tertiary hospitals are the main health institutions for managing hip fractures, while tertiary hospitals manage more intractable and rare diseases referred from primary and secondary care. Finally, for the respondent-level sampling, more than 5 eligible orthopedists in the selected hospitals were invited to answer the questionnaire.

### 2.2. Questionnaires

A standardized questionnaire was administered by a well-trained research team. This questionnaire collected information on basic characteristics such as age, working seniority, hospital level, and residence, with two other parts that include perioperative treatment and postoperative care. The following variables were surveyed: monthly number of admissions for intertrochanteric fractures (1–10, 11–30, 31–50, 51–100, and >100), preoperative waiting time (<12, 12–24, 24–48, 48–72, and >72 h), length of hospitalization (1–3, 4–7, 8–14, and >14 days), rationale for conservative treatment (comorbidities and contraindications, SES, and other reasons), and primary cause of death (pulmonary embolism, cardiovascular disease, cerebrovascular accident, infection, and other causes). Except for the close-ended, multiple-choice responses, questions pertaining to the rationale for conservative treatment and the leading cause of death could be given more than one answer, including “Other” with an open response. We ensured that the sequence of questions was logical and used a web-based platform for ease in responding. This questionnaire was not significantly associated with individual patients of interest but focused on orthopedic surgeons, who were required to complete the questionnaire within the past month/year to minimize recall bias.

The 30 provinces were categorized into six regions according to the method used by the National Bureau of Statistics of China: Eastern (1 city (Shanghai) and 6 provinces (Shandong, Jiangsu, Zhejiang, Fujian, Anhui, and Jiangxi)), Northern (2 cities (Beijing and Tianjin) and 3 provinces (Hebei, Shanxi, and Inner Mongolia)), South-Central (6 provinces (Henan, Hubei, Hunan, Guangdong, Guangxi, and Hainan)), South-Western (1 city (Chongqing) and 3 provinces (Sichuan, Guizhou, and Yunnan)), North-Eastern (3 provinces (Liaoning, Jilin, and Heilongjiang)), and North-Western (5 provinces (Shaanxi, Gansu, Qinghai, Ningxia, and Xinjiang)). Individuals living in Taiwan, Hong Kong, Macao, and Tibet were not included in this survey [26]. These regions have different SES, climate and terrain, and living habits, representing the basic conditions across the country.

The questionnaires were distributed by email to the selected hospitals. After confirming with the selected surgeons, the emails were sent 3 times each at 1 week apart, and the web-based questionnaires were concurrently sent to the surgeons through mobile. The total collection period spanned 2 months. All questionnaires and emails were reviewed and subjected to logical examination, intrasubject comparison, and essential enquiry.

### 2.3. Statistical Analysis

Categorical data were summarized as percentages. Odds ratios (OR) and 95% confidence intervals (CI) were used to determine statistical differences. The proportion of the options in each region was compared using the chi-square (*χ*^2^) test. We created a histogram and choropleth map of the monthly number of admissions using Excel 2016 to compare the distribution characteristics. The analysis was divided into three parts: (i) monthly number of admissions for intertrochanteric fractures; (ii) comparison of the perioperative treatment and postoperative care; and (iii) identification of the rationale for conservative treatment and the leading causes of death. All statistical analysis was performed using SPSS (SPSS for windows, version 22.0).

## 3. Results

### 3.1. Participants

Of the 1101 emails and questionnaires sent, we excluded 36 due to insufficient responses, missing questionnaires, or logical errors. Thus, 1065 (96.7%) valid responses, including 988 (92.8%) mobile phone-based questionnaires and 77 (7.2%) emails, were collected and included in the analysis. During the study period, the majority of orthopedic surgeons were aged over 30 years and had more than 5 years of experience. In addition, approximately half of them were from secondary hospitals, consistent with the sampling strategy we mentioned above (Table 1).

### 3.2. Monthly Number of Admissions for Intertrochanteric Fractures

Although a steep rise of intertrochanteric fractures was expected, the distribution of monthly admissions in different regions and different levels of hospitals was relatively stable. There was a significant difference in the monthly admissions for intertrochanteric fractures according to the geographic areas (*α*^2^ = 47.454, *p* < 0.001). The total number of admissions was relatively lower in the North-Western and South-Western regions. The monthly number of admissions was associated with SES.

**Figure 1:**
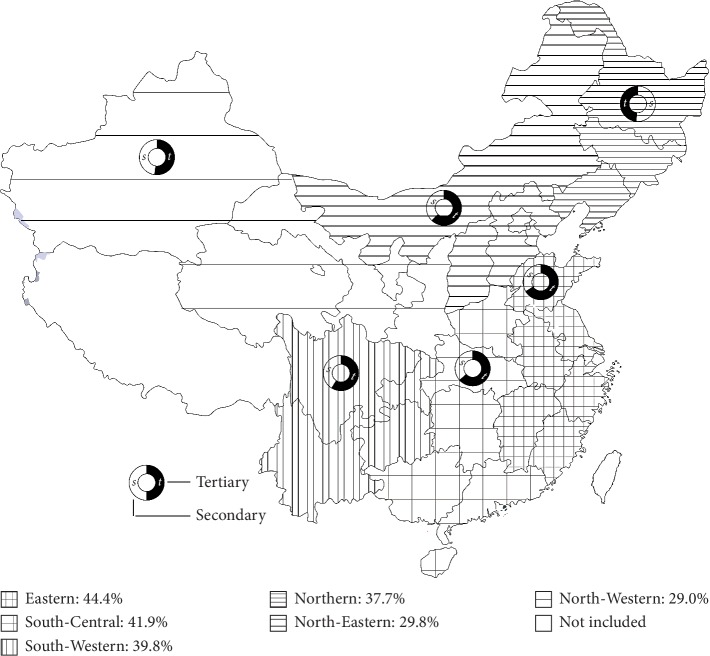
Comparison of monthly number of admissions of intertrochanteric femoral fractures in different areas and levels of hospital in the national investigation of hip fracture care survey.

**Figure 2:**
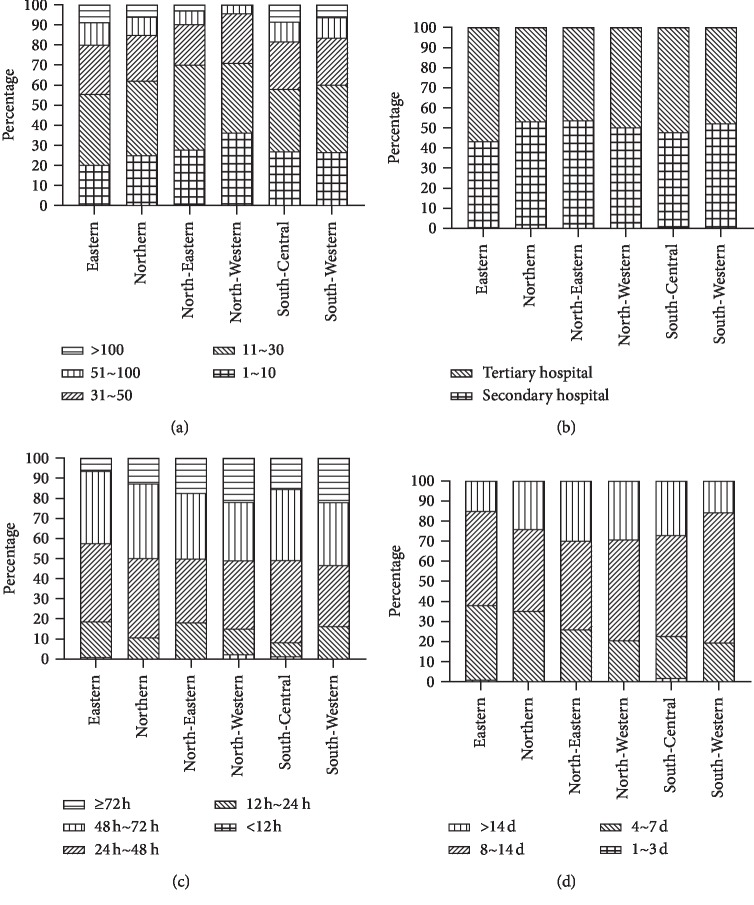
Characteristic variations in intertrochanteric femoral fractures in different areas of China. The distribution of the (a) percentage of monthly admissions, (b) proportion of hospitalizations to secondary and tertiary hospitals, (c) percentage of preoperative waiting time, and (d) length of hospitalization.

We also analyzed the proportion of hospitals with over 30 admissions for intertrochanteric fractures per month. The North-Western and North-Eastern had the lowest percentage (29.1% [48 of 165] and 29.8% [31 of 104], respectively), followed by the Northern (37.7%; 63 of 167), South-Western (39.8%; 51 of 128), South-Central (41.9%; 90 of 215), and the Eastern regions (44.4%; 127 of 286) (Table 2 and Figures 1 and 2(a)).

The proportion of hospitalizations to secondary hospitals and tertiary hospitals was further analyzed. Among the hospitals with over 30 admissions per month, secondary hospitals accounted for more than half in the North-Eastern regions (51.6%; 16 of 31), followed by the North-Western region (47.9%; 23 of 48). The proportion of secondary hospital was significantly lower in the Eastern (33.9%; 43 of 127), South-Central (36.7%; 33 of 90), and the Northern region (38.1%; 24 of 63) (Figures 1 and 2(b)).

### 3.3. Preoperative Waiting Time and Postoperative Length of Hospitalization

Preoperative waiting time and postoperative hospitalization are critical determinants of mortality, comorbidity, and recovery [27]. Accordingly, we analyzed their distribution among different regions and different levels of hospitals.

The most frequent preoperative waiting time and hospitalization period was ≥12 hours (99.1%) and >3 days (99.3%), respectively. The highest percentage of long preoperative waiting time (≥72 h) was in the South-Western region (21.9%; 28 of 128) (reference: Eastern region (6.3% [18 of 286]; OR: 4.17; 95% CI: 2.21–7.87; *p* < 0.001)). Meanwhile, the highest percentage of short hospitalization period (<7 days) was in the Eastern region (38.1%; 109 of 286), while the lowest was in the South-Western (19.5% [25 of 128]; OR: 2.54; 95% CI: 1.54–4.17; *p* < 0.001) (Table 2; Figures 2(c) and 2(d)).

A longer preoperative waiting time (≥72 h) occurred more frequently in secondary hospitals compared with tertiary hospitals (19.4% [101 of 522] versus 7.9% [43 of 543]; OR: 2.79; 95% CI: 1.91–4.08; *p* < 0.001). Additionally, a shorter hospitalization period (<7 days) was more likely in tertiary hospitals compared with secondary hospitals (37.2% [202 of 543] versus 19.4% [101 of 522]; OR: 2.47; 95% CI: 1.87–3.26; *p* < 0.001). Overall, tertiary hospitals appeared to have a shorter preoperative waiting time and a shorter hospitalization period (Table 3).

### 3.4. Rationale for Conservative Treatment and Leading Causes of Perioperative Death

Some patients with advanced illness and high risks would benefit from conservative treatment, which is the main reason of comorbidities on fracture-related death as well [28]. Thus, establishing the appropriate rationale for conservative treatment and identifying the leading causes of death in this population are crucial. The most common rationale for conservative treatment was “comorbidities and contraindications” (72.5%; 772/1065). Interestingly, the frequency of SES as a rationale for conservative treatment was relatively higher in the Western regions, particularly in the South-Western (28.1%; 36 of 128), than that in the Eastern region (7.7% [22 of 286]; OR: 4.70; 95% CI: 2.63–8.40; *p* < 0.001). Complications, including “pulmonary embolism” (83.1%; 885 of 1065), “cardiovascular disease” (50.5%; 538 of 1065), and “cerebrovascular accident” (24.4%; 260 of 1065) were identified as the leading causes of perioperative death (Table 2).

## 4. Discussion

This national orthopedic surgeon-oriented survey showed a remarkable variation in the number of monthly admissions, preoperative waiting time, length of hospitalization, and rationale for conservative treatment according to the geographic division and hospital level in China.

Area-level SES has been reported to be associated with the incidence of hip fractures. Guilley et al. reported that in Switzerland, those with medium income have a lower hip fracture incidence compared with those with lower income [29]. However, another study by Reyes et al. found that the incidence of hip fracture was lower in the lowest SES quintiles [30]. Holloway et al. and Bacon and Hadden reported that individuals with higher income had a lower risk of hip fracture [21, 31]. The difference in the results among these studies could be attributed to the different definitions of SES, climate and environment of each city, and the age/sex composition. As there was no similar study in China, a country with a vast diversity in SES, lifestyle, and healthcare policy, we conducted this pilot study to provide a dataset that can be used to draft national health policies for the prevention and management of hip fracture, particularly in the elderly.

Unlike previous studies, our findings focused on monthly admissions instead of intertrochanteric fracture incidence. The absolute number of admissions is more accurate than the incidence due to the huge floating population in big cities. We found an apparent geographic variation in the proportion of hospitals with over 30 admissions per month (Table 2 and Figure 1). The North-Western and North-Eastern had the lowest percentage of these hospitals (29.0% and 29.8%, respectively), while the Eastern region had the highest percentage (44.4%). The Eastern region has a higher gross domestic product than the Western region. Accordingly, our data show that the monthly admissions of intertrochanteric fracture were lower in the low SES region. There are several possible explanations for the diversity. First, socioeconomic development was relatively lower in the Western regions, which may contribute to fewer tertiary hospitals and scattered medical resources. Second, the residents in the Western regions might not seek better medical treatment due to inconvenient traffic and poverty. Surgical treatment, including osteosynthesis and arthroplasty, serves as the gold standard modality in the elderly to reduce the mortality [32]. Meanwhile, palliative treatment is the only option for the critically ill [33].

Previous studies have examined the reasons for long preoperative waiting time and found the main reasons are limited number of operating rooms, time-consuming examinations in patients with comorbidities, and anticoagulant agents [34], which significantly increase the risk of death and pressure sores [35–37]. Several studies have established the association between preoperative waiting time and subsequent morbidity and mortality in elderly patients [38, 39]. Performing surgery within 48 hours of hospital admission, regardless of the surgical approach, is crucial in improving outcomes and mortality. In a prospective observational study of 5683 male patients aged over 65 years with hip fractures, an operative delay beyond 4 days caused a higher mortality risk [38]. Additionally, previous meta-analysis of 250,000 patients showed an absolutely increased risk of 30-day and 1-year mortality as a result of surgical delay [39–41]. In Japan, improvements in medical policy shortened the hospitalization period from 48.1 days in 2004 to 36.8 days in 2014 [34, 42]. However, the waiting time in Japan was longer than that in other countries [35, 43, 44], which might be attributed to the difference in population density and medical policy. Findings from the current study also showed a remarkable variation in the preoperative waiting time and hospitalization period according to the geographic areas and hospital level. Preoperative waiting time was markedly longer in the Western regions (North-Western and South-Western) and in secondary hospitals than that in the Eastern region and tertiary hospitals. This finding may be due to the inadequate number of operating rooms and limited medical facilities in secondary hospitals and Western regions in China.

The most predominant rationale for the palliative treatment of intertrochanteric fracture was comorbidities and contraindications in the higher SES regions and social and economic status in the lower SES regions. The influence of SES on the outcomes of intertrochanteric fracture has not been evaluated in many studies in developed countries; however, it might play a fundamental role in China. Patients in remote areas such as the Western region were more likely to choose palliative treatment due to poverty. However, comorbidities and contraindications were the first concerns in developed areas such as the Eastern region. Pulmonary embolism, cardiovascular disease, and cerebrovascular accident were identified as the primary causes of death across China (Table 2).

This study has several strengths. The NIH-FC represented a national sample of secondary and tertiary hospitals. The high response rate, attributed to the two different strategies used, could reduce responder bias. Further, the participants were doctors, instead of patients, and were selected via strict multistage probability sampling procedure [25, 45]. Thus, the data from the NIH-FC allowed us to describe the multiple aspects of intertrochanteric fractures according to geographic variations and hospital levels. Our findings will be helpful in formulating health-promotion strategies and medical policy and ultimately improving health care across the country.

However, this study also has some limitations. First, we used survey data, and thus, our findings might not be generalizable to the entire country. Second, the responses might be subjective. Although we used logical examination and repeated questionnaires to minimize bias, the inherent drawback of an epidemiological study should not be ignored. Finally, there was no concrete score to evaluate SES and hospital level in China, such as the Accessibility/Remoteness Index of Australia and Index of Relative Socioeconomic Advantage and Disadvantage scores in Australia [21]. Thus, we used income from the National Bureau of Statistics of China as a measure of SES.

## 5. Conclusions

This study showed apparent geographic variations in monthly admissions of intertrochanteric fractures, preoperative waiting time, and hospitalization periods across China. Overall, the monthly number of admissions was relatively lower in the Northern region. Preoperative waiting time and length of hospitalization was shorter in the Eastern region and tertiary hospitals.

## Figures and Tables

**Table 1:** Characteristics of subjects in the national investigation of hip-fracture care survey.

Characteristics	Eastern	Northern	North-Eastern	North-Western	South-Central	South-Western	Total
Individuals	286	167	104	165	215	128	1065

Age (years)							
≦30	15 (5.3)	7 (4.2)	9 (8.7)	12 (7.3)	24 (11.2)	13 (10.2)	80 (7.5)
31–40	206 (72.0)	119 (71.3)	62 (60.0)	98 (59.4)	143 (66.5)	80 (62.5)	708 (66.5)
41–50	61 (21.3)	40 (24.0)	32 (30.8)	50 (30.3)	39 (18.1)	33 (25.8)	255 (24.0)
51–60	4 (1.4)	1 (0.5)	1 (0.5)	5 (3.0)	8 (3.7)	2 (1.5)	21 (2.0)
>60	0	0	0	0	1 (0.5)	0	1 (0.0)

Working seniority (years)							
<5	15 (5.2)	3 (1.8)	8 (7.7)	10 (6.1)	18 (8.4)	10 (7.8)	64 (6.0)
5–10	90 (31.5)	52 (31.1)	24 (23.1)	43 (26.1)	77 (35.8)	39 (30.5)	325 (30.5)
11–20	139 (48.6)	92 (55.1)	60 (57.7)	87 (52.7)	87 (40.5)	60 (46.9)	525 (49.3)
>20	42 (14.7)	20 (12.0)	12 (11.5)	25 (15.1)	33 (15.3)	19 (14.8)	151 (14.2)

Hospital level							
Secondary hospital	124 (43.4)	89 (53.3)	56 (53.8)	83 (50.3)	103 (47.9)	67 (52.3)	522 (49.0)
Tertiary hospital	162 (56.6)	78 (46.7)	48 (46.2)	82 (49.7)	112 (52.1)	61 (47.7)	543 (51.0)

*Note*. Except where indicated otherwise, values are the number (weighted percentage).

**Table 2:** Distribution of characteristic variables on the treatment of intertrochanteric fractures.

Characteristics	Eastern	Northern	North-Eastern	North-Western	South-Central	South-Western
Monthly number of cases						
1–10	58 (20.3)	42 (25.1)	29 (27.9)	60 (36.4)	58 (27.0)	34 (26.6)
11–30	101 (35.5)	62 (37.1)	44 (42.3)	57 (34.5)	67 (31.1)	43 (33.6)
31–50	70 (24.5)	38 (22.8)	21 (20.2)	41 (24.9)	51 (23.7)	30 (23.4)
51–100	32 (11.2)	15 (9.0)	7 (6.7)	7 (4.2)	21 (9.8)	13 (10.2)
>100	25 (8.7)	10 (6.0)	3 (2.9)	0	18 (8.4)	8 (6.2)

Preoperative waiting time (h)						
<12	3 (1.0)	0	0	4 (2.4)	3 (1.4)	0
12–24	51 (17.8)	18 (10.8)	19 (18.3)	21 (12.7)	15 (7.0)	21 (16.4)
24–48	111 (38.9)	66 (39.5)	33 (31.7)	56 (34.0)	88 (40.9)	39 (30.4)
48–72	103 (36.0)	62 (37.1)	34 (32.7)	48 (29.1)	76 (35.3)	40 (31.3)
>72	18 (6.3)	21 (12.6)	18 (17.3)	36 (21.8)	33 (15.4)	28 (21.9)

Length of hospitalization period (d)						
1–3	3 (1.0)	0	0	0	4 (1.9)	0
4–7	106 (37.1)	59 (35.3)	27 (26.0)	34 (20.6)	45 (20.9)	25 (19.5)
8–14	134 (46.9)	68 (40.7)	46 (44.2)	83 (50.3)	108 (50.2)	83 (64.9)
>14	43 (15.0)	40 (24.0)	31 (29.8)	48 (29.1)	58 (27.0)	20 (15.6)

Rationale for conservative treatment						
Comorbidities and contraindications	232 (81.1)	140 (83.8)	76 (73.1)	102 (61.8)	133 (61.9)	89 (69.5)
Social and economic status	22 (7.7)	17 (10.2)	16 (15.4)	42 (25.5)	56 (26.0)	36 (28.1)
Other reasons	32 (11.2)	10 (6.0)	12 (11.5)	21 (12.7)	26 (12.1)	3 (2.4)

The most common causes of death						
Pulmonary embolism	250 (87.4)	135 (80.8)	92 (88.5)	128 (77.6)	174 (80.9)	106 (82.8)
Cardiovascular disease	140 (49.0)	73 (43.7)	49 (47.1)	90 (54.5)	116 (54.0)	70 (54.7)
Cerebrovascular accident	83 (29.0)	50 (30.0)	23 (22.1)	35 (21.2)	39 (18.1)	30 (23.4)
Infection	30 (10.5)	14 (8.4)	13 (12.5)	20 (12.1)	34 (15.8)	14 (10.9)
Other causes	24 (8.4)	10 (6.0)	3 (2.9)	5 (3.0)	22 (10.2)	8 (6.3)

*Note*. Except where indicated otherwise, values are the number (weighted percentage).

**Table 3:** Diversity of preoperative waiting time and length of hospitalization period in different levels of hospital.

Characteristics	Secondary hospital	Tertiary hospital	Total
Individuals	522	543	1065

Preoperative waiting time (h)			
<12	7 (1.3)	3 (0.6)	10 (0.9)
12–24	35 (6.7)	68 (12.5)	165 (15.5)
24–48	177 (33.9)	214 (39.4)	383 (36.0)
48–72	202 (38.7)	215 (39.6)	363 (34.1)
>72	101 (19.4)	43 (7.9)	144 (13.5)

Length of hospitalization period (d)			
1–3	3 (0.6)	4 (0.7)	7 (0.7)
4–7	98 (18.8)	198 (36.5)	296 (27.8)
8–14	236 (45.2)	286 (52.7)	522 (49.0)
>14	185 (35.4)	55 (10.1)	240 (22.5)

*Note*. Except where indicated otherwise, values are the number (weighted percentage).

## Data Availability

All data are available on request.
